# Assessing infection prevention and control structures in German hospitals after the COVID-19 pandemic using the WHO infection prevention and control assessment framework (IPCAF): results from 660 hospitals and comparison with a pre-pandemic survey

**DOI:** 10.1186/s13756-024-01465-7

**Published:** 2024-09-13

**Authors:** Ferenc Darius Rüther, Alexander Gropmann, Sonja Hansen, Michael Behnke, Christine Geffers, Seven Johannes Sam Aghdassi

**Affiliations:** 1 Institute of Hygiene and Environmental Medicine, Charité –Universitätsmedizin Berlin, corporate member of Freie Universität Berlin and Humboldt-Universität zu Berlin, Berlin, Germany; 2 National Reference Center for Surveillance of Nosocomial Infections, Berlin, Germany; 3 BIH Biomedical Innovation Academy, BIH Charité Digital Clinician Scientist Program, Berlin Institute of Health at Charité – Universitätsmedizin Berlin, Berlin, Germany

**Keywords:** Infection control, IPCAF, Survey, Surveillance, Infection control structures

## Abstract

**Background:**

The WHO Infection Prevention and Control Assessment Framework (IPCAF) is a standardized tool to assess infection prevention and control (IPC) structures in healthcare facilities. The IPCAF reflects the eight WHO core components (CC) of IPC. Besides facility self-assessment, the IPCAF can be used for national surveys, and repeated usage can aid in describing trends concerning IPC structures. A previous survey in over 700 German hospitals conducted in 2018, yielded an overall high IPC level in participating hospitals, albeit with potentials for improvement. In 2023, the survey was repeated to describe once again the state of IPC implementation in German hospitals and compare findings to data from 2018.

**Methods:**

The German National Reference Center for the Surveillance of Nosocomial Infections (NRC) invited 1,530 German acute care hospitals participating in the national surveillance network “KISS”, to complete a translated online version of the IPCAF between October 2023 and January 2024. The questionnaire-like nature of the IPCAF, where each answer corresponds to a number of points, allows for calculating an overall IPC score. Based on the overall score, hospitals were allocated to four different IPC levels: inadequate (0–200), basic (201–400), intermediate (401–600), and advanced (601–800). Aggregated scores were calculated and compared with results from 2018.

**Results:**

Complete datasets from 660 hospitals were received and analyzed. The median overall IPCAF score was 692.5 (interquartile range: 642.5–737.5), with 572 hospitals (86.6%) classified as advanced, and 87 hospitals (13.2%) as intermediate. One hospital (0.2%) fell into the basic category. The overall median score was virtually unchanged when compared to 2018 (690; data from 736 hospitals). The median score for the CC on workload, staffing and bed occupancy was markedly higher (85 vs. 75), whereas the median score for the CC on multimodal strategies was slightly lower than in 2018 (75 vs. 80).

**Conclusions:**

Repeated assessments of IPC structures at the national level with the IPCAF are feasible and a means to gain insights into the evolution of IPC structures. When comparing aggregated scores, a stable and high level of IPC key aspects in Germany was observed, with improvements over time in IPC indicators related to workload and staffing.

**Supplementary Information:**

The online version contains supplementary material available at 10.1186/s13756-024-01465-7.

## Background

Successful and sustainable infection prevention and control (IPC) is predicated on the existence of dedicated structures, strategies and processes. Identifying and implementing these in any sector of healthcare is crucial, albeit challenging. To provide an orientation on the matter, the World Health Organization (WHO) published a guideline document that outlines core components (CC) for successful IPC programs [1]. The guidelines make a distinction between IPC implementation at the national and the facility level. To complement the guideline document and provide facilities with a tool to systematically assess the degree to which IPC structures and processes are implemented, the WHO released the Infection Prevention and Control Assessment Framework (IPCAF) [2].

The IPCAF is primarily intended for facility self-assessment. However, it can also serve as a basis for surveys on a larger scale, e.g. to assess key IPC structures across hospitals in a given country. Shortly after the release of the IPCAF, in the fall of 2018, we used the tool for a national survey and reported an analysis of IPCAF data from 736 German acute care hospitals [3]. Overall, the 2018 survey revealed an advanced level of IPC in the participating German hospitals. Worldwide, a global survey from the WHO conducted in 2019, also found an overall advanced level of IPC but with considerable variability between geographical regions and lower scores in low and middle income countries [4]. The feasibility of utilizing the IPCAF to conduct assessments on IPC structures in hospitals through national surveys, has also been demonstrated in various other publications. Results from surveys similar to ours in 2018, were published from Indonesia (n = 355 hospitals), China (n = 222), Turkey (n = 68), Austria (n = 65), Japan (n = 59), Cote d’Ivoire (n = 30), Bangladesh (n = 11), Pakistan (n = 5) and Ghana (n = 3) [5–13]. These publications provided valuable insights into the IPC situation in the respective countries and also helped to establish IPCAF as a popular tool for IPC assessment.

However, an integral part of the WHO’s intended use of the IPCAF, is its repeated use to uncover developments and trends, potentially attributable to changes that were implemented as a result of previously observed deficits [2]. In the context of utilizing the IPCAF for national surveys, repeated usage may provide insights into potential developments at the national level. To the best of our knowledge, results of repeated national usage of the IPCAF have not been published yet. Accordingly, we decided to use the IPCAF for another survey in German hospitals five years after its first application. We aimed to describe once again the state IPC implementation in a convenience sample of German hospitals, and to compare current data to results from 2018, with the goal of deriving insights into potential large-scale changes. Indirectly, the repeated usage of the IPCAF before and after the COVID-19 pandemic, may also yield insights into developments potentially linked to the pandemic.

## Methods

The IPCAF is a questionnaire for healthcare facilities and consists of eight sections corresponding to the eight CC of IPC for healthcare facilities defined in the above-mentioned WHO guideline [1, 14]. The eight sections of the IPCAF are:

- IPC program (CC1)
- IPC guidelines (CC2)
- IPC education and training (CC3)
- Healthcare-associated infection (HAI) surveillance (CC4)
- Multimodal strategies for implementing IPC interventions (CC5)
- Monitoring/audit of IPC practices and feedback (CC6)
- Workload, staffing and bed occupancy (CC7)
- Built environments, materials and equipment for IPC (CC8)

The IPCAF follows an approach, whereupon each answer to every question corresponds to a score. Scores are summed up for every CC. Finally, the scores of all eight CC, each with a maximum score of 100, are added up, to determine the overall IPCAF score. Depending on the final score, healthcare facilities are grouped into four different IPC categories:

- 0–200 points: inadequate
- 201–400 points: basic
- 401–600 points: intermediate
- 601–800 points: advanced

As in 2018, the IPCAF was distributed as the annual survey of the German nosocomial infection surveillance system “KISS” (Krankenhaus-Infektions-Surveillance-System) to participating hospitals. KISS is organized by the German National Reference Center for Surveillance of Nosocomial Infections (NRC) and represents the national network for HAI surveillance in Germany. KISS is divided into distinct modules that pertain to different settings or patient populations (e.g., intensive care units, surgical patients, neonates), types of infections or multidrug-resistant organisms as well as other IPC indicators (e.g., alcoholic hand rub consumption). While HAI surveillance is mandated by law for hospitals in Germany, the exact means of conducting surveillance are not stipulated and participation in KISS is ultimately voluntary. On October 10, 2023, the NRC sent a link to a survey webpage (LimeSurvey Community Edition Version 5.2.7) with the German IPCAF translation to 1,530 acute care hospitals in Germany. Of note, as per the most recent available version of the German hospital register, there are 1,827 hospitals in Germany [15]. Recipients of the invitation were the respective hospital focal persons for surveillance listed in the KISS database (primarily IPC nurses or physicians). All data were entered online. Minor amendments were made to the German IPCAF translation used in 2018, to increase the clarity of some questions and update expired online links. The translated version used for the 2023 survey can be found in the online supplement (Additional file 1).

Data was entered on a voluntary basis and could be submitted until January 15, 2024. Participation was incentivized by making survey participation a criterion for obtaining the annual “KISS certificate”, however, obtaining the certificate without survey participation was still possible, if other criteria were met by the hospitals. After submission, the results were automatically transferred to the NRC. As per agreement with the survey participants, the received data were not linked to surveillance data or other data, such as alcoholic hand rub consumption of the participating hospitals. Likewise, datasets from 2023 and 2018 were not matched, which rules out analyzing data of hospitals that participated in both surveys separately. These decisions were made to decrease the potential of the IPCAF survey to be perceived as potentially compromising by participating hospitals, and thereby reduce the potential for wishful reporting.

In alignment with our previous reports of IPCAF data, we excluded incomplete datasets from the data analysis (i.e., incomplete datasets were disregarded in their entirety) [3]. A descriptive analysis of the overall IPCAF score, the CC and selected questions of interest was conducted by the NRC. Furthermore, aggregated data of 2023 was compared with aggregated results from 2018. Results from 2018 were comprehensively reported in a previous publication [3], and will only be repeated when crucial for the proper interpretation of the results from 2023. Data analysis and graphical representation were carried out with Microsoft Excel 2016.

## Results

**Fig. 1:**
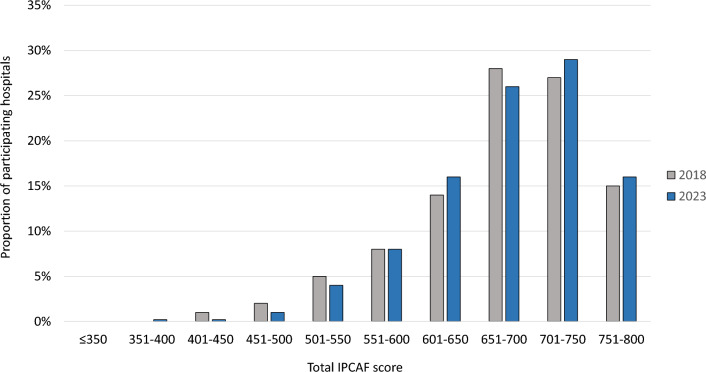
Proportion of hospitals with different IPCAF scores in 2023 vs. 2018. Data from 660 German acute care hospitals in 2023 and 736 hospitals in 2018. Abbreviations: *IPCAF* Infection Prevention and Control Assessment Framework

In total, 1,530 German acute care hospitals were invited to participate. Of these, 755 (49.3%) transmitted their responses to the NRC. Due to missing data, 95 (12.6%) were excluded from the analysis. Accordingly, the IPCAF was fully completed and submitted to the NRC by 660 hospitals (response rate of 43.1%). When applying the above-mentioned IPC categories, 572 hospitals (86.6%) fell into the advanced category, 87 hospitals (13.2%) were allocated to the intermediate category and one hospital (0.2%) did not exceed the basic category. No hospital had a score that would correspond to an inadequate IPC level. The median overall IPCAF score was 692.5 (interquartile range: 642.5–737.5). When looking at the results from 2018, the distribution of hospitals by total IPCAF score remained nearly unchanged (Fig. 1).

**Fig. 2:**
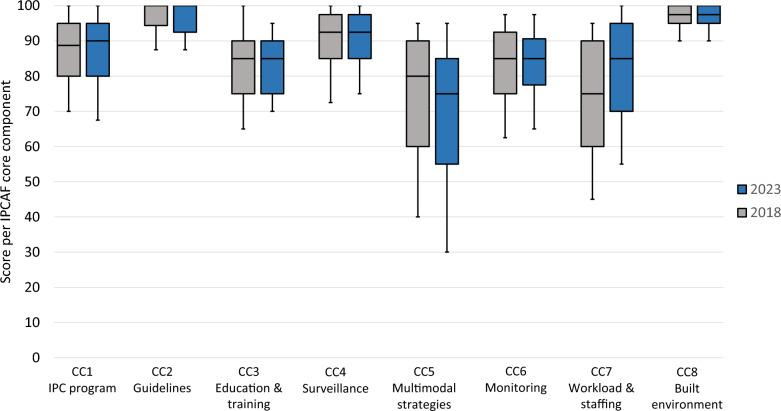
Boxplots displaying the median and range of IPCAF core component scores in 2023 vs. 2018. Data from 660 German acute care hospitals in 2023 and 736 hospitals in 2018. The boxplots display the distribution of scores per core component. The horizontal lines in the box represent the median, the top and bottom of the box represent the interquartile range, the whiskers illustrate the tenth and 90th percentile. Abbreviations: *CC* Core component, *IPCAF* Infection Prevention and Control Assessment Framework

Regarding the individual components, the lowest median (75) and mean (68.5) scores were observed for CC5 (multimodal strategies), followed by CC7 (workload, staffing, ward design and bed occupancy) (Table 1). In contrast, guidelines (CC2) and environment/infrastructure (CC8) had the highest median (100) and mean (96.3) scores, respectively. The range of scores per component (range between the tenth and the 90th percentile) was broadest for CC5 (30–95) and CC7 (55–100), and narrowest for CC8 (90–100) and CC2 (87.5–100). When compared to results from 2018, the largest increase and decrease in points was recorded for CC7 (median score + 10) and CC5 (median score -5), respectively. Overall, a high degree of consistency was observed with regard to both median and range (Fig. 2).

**Table 1:** Distribution of results of the total IPCAF score and scores per core component

Component	Score					
	Q10	Q25	**Q50**	Q75	Q90	Mean
CC1: Infection Prevention and Control (IPC) program	70	80	**90**	95	100	85.7
CC2: IPC guidelines	87.5	92.5	**100**	100	100	95.9
CC3: IPC education and training	70	75	**85**	90	95	82.9
CC4: Healthcare-associated infection (HAI) surveillance	75	85	**92.5**	97.5	100	89.2
CC5: Multimodal strategies for implementation of IPC interventions	30	55	**75**	85	95	68.5
CC6: Monitoring/audit of IPC practices and feedback	65	77.5	**85**	90.6	97.5	83.3
CC7: Workload, staffing and bed occupancy	55	70	**85**	95	100	81.5
CC8: Built environment, materials and equipment for IPC at the facility level	90	95	**97.5**	100	100	96.3
Total	589.8	642.5	**692.5**	737.5	765	683.3

Data from 660 German acute care hospitals in 2023. Abbreviations: *IPCAF* Infection Prevention and Control Assessment Framework; *CC* core component; *Q10* tenth percentile; *Q25* first quartile; *Q50* median (bold numbers); *Q75* third quartile; *Q90* 90th percentile

For the sake of a concise presentation, a focus will be placed on multimodal strategies (CC5) as the CC with the lowest median score, and workload, staffing and bed occupancy (CC7) as the CC with the highest increase in the median score. A comprehensive display of all questions and answers can be found in online supplement (Additional file 2).

Regarding CC5, almost a third of all responding hospitals reported not employing a multidisciplinary team to implement IPC multimodal strategies (30.2%), or did not regularly cooperate with colleagues from quality improvement and patient safety to develop and promote IPC multimodal strategies (29.2%). Further details on CC5 are illustrated in Table 2.

**Table 2:** Results per element of multimodal strategies for implementation of IPC interventions (IPCAF core component 5)

Element	Answer	Number (%) IPCAF 2023
System change	Element not included in multimodal strategies	143 (21.7)
	Interventions to ensure the necessary infrastructure and continuous availability of supplies are in place	216 (32.7)
	Interventions to ensure the necessary infrastructure and continuous availability of supplies are in place and addressing ergonomics and accessibility, such as the best placement of central venous catheter set and tray	301 (45.6)
Education and training	Element not included in multimodal strategies	44 (6.7)
	Written information and/or oral instruction and/or e-learning only	355 (53.8)
	Additional interactive training sessions (includes simulation and/or bedside training)	261 (39.5)
Monitoring and feedback	Element not included in multimodal strategies	63 (9.5)
	Monitoring compliance with process or outcome indicators (for example, audits of hand hygiene or catheter practices)	186 (28.2)
	Monitoring compliance and providing timely feedback of monitoring results to healthcare workers and key players	411 (62.3)
Communications and reminders	Element not included in multimodal strategies	125 (18.9)
	Reminders, posters, or other advocacy/awareness-raising tools to promote the intervention	352 (53.4)
	Additional methods/initiatives to improve team communication across units and disciplines (for example, by establishing regular case conferences and feedback rounds)	183 (27.7)
Safety climate and culture change	Element not included in multimodal strategies	271 (41.1)
	Managers/leaders show visible support and act as champions and role models, promoting an adaptive approach and strengthening a culture that supports IPC, patient safety and quality	271 (41.1)
	Additionally, teams and individuals are empowered so that they perceive ownership of the intervention (for example, by participatory feedback rounds)	118 (17.8)

Data from 660 German acute care hospitals in 2023. Abbreviations: *IPCAF* Infection Prevention and Control Assessment Framework

The most pronounced changes were observed for CC7 (workload, staffing and bed occupancy), with an increase of 17.2 percentage points in hospitals assessing staffing levels (83.8% in 2023 vs. 66.6% in 2018), an increase of 17.2 percentage points in hospitals maintaining (to some extent) an established ratio of healthcare workers to patients (92.7% vs. 75.5%), and an increase of 18.4 percentage points in hospitals maintaining a system to act on the results of the staffing needs assessments (83.8% vs. 65.4%). Table 3 displays the full results of CC7.

**Table 3:** Results from IPCAF core component 7 (workload, staffing and bed occupancy)

		Number (%) IPCAF 2023
Question 1	Are appropriate staffing levels assessed in your facility according to patient workload using national standards or a standard staffing needs assessment tool such as the WHO Workload indicators of staffing need method?	
	No	107 (16.2)
	Yes	553 (83.8)
Question 2	Is an agreed (that is, WHO or national) ratio of health care workers to patients maintained across your facility?	
	No	48 (7.3)
	Yes, for staff in less than 50% of units	43 (6.5)
	Yes, for staff in more than 50% of units	215 (32.6)
	Yes, for all health care workers in the facility	354 (53.6)
Question 3	Is a system in place in your facility to act on the results of the staffing needs assessments when staffing levels are deemed to be too low?	
	No	107 (16.2)
	Yes	553 (83.8)
Question 4	Is the design of wards in your facility in accordance with international standards regarding bed capacity?	
	No	42 (6.4)
	Yes, but only in certain departments	215 (32.6)
	Yes, for all departments (including emergency department and pediatrics)	403 (61.0)
Question 5	Is bed occupancy in your facility kept to one patient per bed?	
	No	10 (1.5)
	Yes, but only in certain departments	44 (6.7)
	Yes, for all units (including emergency departments and pediatrics)	606 (91.8)
Question 6	Are patients in your facility placed in beds standing in the corridor outside of the room (including beds in the emergency department)?	
	Yes, more frequently than twice a week	70 (10.6)
	Yes, less frequently than twice a week	104 (15.8)
	No	486 (73.6)
Question 7	Is adequate spacing of > 1 m between patient beds ensured in your facility?	
	No	16 (2.4)
	Yes, but only in certain departments	134 (20.3)
	Yes, for all departments (including emergency department and pediatrics)	510 (77.3)
Question 8	Is a system in place in your facility to assess and respond when adequate bed capacity is exceeded?	
	No	18 (2.7)
	Yes, this is the responsibility of the head of department	237 (35.9)
	Yes, this is the responsibility of the hospital administration/management	405 (61.4)

Data from 660 German acute care hospitals in 2023. Abbreviations: *IPCAF* Infection Prevention and Control Assessment Framework; *WHO* World Health Organization

## Discussion

To the best of our knowledge, this is the first study re-assessing IPC structures in a large number of hospitals through the IPCAF. The number of participating German acute care hospitals (n = 660, response rate of 43.1%) was around 10% lower than in 2018 (n = 739, response rate of 50.2%) although the method of distribution (via the German KISS network) and participation (via an online survey) did not change. Given the length of the questionnaire as well as ongoing challenges due to stretched IPC resources in many hospitals, the response rate and overall participation are nevertheless remarkably high and document a good uptake of the IPCAF as an IPC assessment tool.

Overall, the median IPCAF score remained almost unchanged (693 vs. 690) with a similar proportion of hospitals achieving an advanced (86.6% vs. 84.5%), intermediate (13.2% vs. 15.1%) or basic (0.2% vs. 0.4%) IPC category. The high degree of concordance between results from 2018 and 2023 is striking given the COVID-19 pandemic occurred between the two surveys. The pandemic saw an increase in attention to certain aspects of IPC. However, our survey indicates that this may not have resulted in structural changes that could have been appreciated by the IPCAF. Similarly, other studies that have investigated the effects of the pandemic on IPC structures and practices, have revealed that the COVID-19 pandemic may have triggered a shift in the allocation of IPC resources rather than all-encompassing improvements [16, 17]. Of note, it is important to acknowledge that IPC structures as documented in the survey 2018, were already at a high level in Germany before the pandemic, leaving less room for improvement, and that the pandemic may have triggered changes that were not captured by the IPCAF.

The median scores of all CC, except CC5 (multimodal strategies for implementation of IPC interventions), were above 75, which reflects that these are generally well-established in Germany. This finding documents the important role of that IPC plays in the German healthcare system, which can for example be seen in the long history of the widely used national HAI surveillance network “KISS” [18].

In 2018, the lowest median score (75) was reported for CC7 (workload, staffing and bed occupancy) [3]. The results from 2023 show a considerable increase in the median score for this CC, especially for questions pertaining to the assessment of staffing levels, healthcare worker to patient ratios and mechanisms to determine staffing needs and react to changes. This could be interpreted as an effect of novel directives and laws with a focus on staffing in the German healthcare system (e.g. the “Nursing Personnel Strengthening Act” [19]), or as a consequence of an increased awareness for the aspects bed occupancy and staff-to-patient ratio that may have developed during the COVID-19 pandemic [20, 21]. Among other things, the Nursing Personnel Strengthening Act provides for a nursing staff ratio for hospitals, which is intended to adjust the level of nursing staff to the nursing workload. This is designed to set a lower limit for nursing staffing levels, which the hospital may not fall below [19]. The broad range of scores for CC7 achieved by hospitals illustrates that the level, to which aspects of workload and staffing are incorporated into IPC practices may vary considerably across German hospitals. Additionally, it is conceivable that deficits observed in the IPCAF 2018 concerning staffing, have motivated some hospitals to better address this aspect.

In 2018, the median score of CC5 (80) was comparatively low, which might have been attributable to the relative novelty of the concept of multimodal strategies for IPC interventions, and potential unfamiliarity of respondents with the concept. One could expect that five years later familiarity with this concept should have been higher and that multimodal strategies would have become more integrated in IPC practices. However, our survey revealed scores for CC5 that were slightly lower than in 2018. Correspondingly, the majority of publications on national IPCAF surveys also identified CC5 as a component with comparatively low scores [5, 7, 10, 11, 13]. It appears that despite the resources and literature already provided on the matter [1, 14, 22, 23], the concept and benefits of multimodal strategies in IPC may still not be fully appreciated by currently implemented IPC standards. While unfamiliarity with the concept of multimodal strategies might be an explanation for the seeming non-progression of multimodal strategies in German IPC practices, it can also be speculated that there could be structural or organizational barriers that render the adoption of multimodal strategies difficult. Such potential underlying barriers should be explored in more focused studies, for instance by means of qualitative surveys on the status quo of multimodal strategies for the implementation of IPC in German hospitals. However, as for CC7, the range of scores for CC5 was particularly broad. This indicates that multimodal strategies may be routinely used by some hospitals, while not being employed or only being employed at a rudimentary level by others, thus documenting variation in how IPC activities are implemented across German hospitals. Further aspects that were identified and discussed in our publication on the IPCAF 2018 as potential targets for improvement (e.g. definition of objectives for IPC programs, application of interactive methods to perform IPC training and feedback of surveillance data, integration of IPC aspects into the training of other specialties [3]), showed no meaningful changes, which documents a continued potential for improvement in certain areas of IPC.

Only a minority of hospitals (38.5%) reported having implemented IPC training for patients or family members (CC3). This corresponds to various reports on the matter that showed that IPC education for patients is not yet widely implemented in many hospitals [24]. Given reports of improved patient hand hygiene through education and subsequent lower infection rates [25–27], it seems appropriate to suggest that patient-directed IPC training programs should be promoted more widely in German hospitals.

Overall, our study demonstrated that usage of the IPCAF on a broad scale is feasible, and that conclusions can be carefully drawn from its repeated application. However, to fully assess the many aspects of IPC a multitude of IPC instruments are necessary. The WHO offers additional tools like the hand hygiene self-assessment framework [28] or assessment tools on infection prevention and control minimum requirements for health care facilities [29, 30]. It is important to see the IPCAF as part of a growing set of global IPC instruments that can be utilized synergistically.

Our study had several limitations. First, data from the participating hospitals constitute a convenience sample and cannot be seen as representative for Germany. All included datasets were from hospitals participating in the German national surveillance network, which may have a greater than average interest in matters of surveillance and IPC. However, due to the high number of participating hospitals (around one third of hospitals listed in the German hospital register [15]) careful extrapolations to the national level appear justifiable. Second, as per agreement with the study participants, data of the survey were not linked at the level of individual hospitals, to other surveillance data or to data from the 2018 IPCAF survey. Accordingly, observed differences in IPCAF scores could be attributable to a different cohort of participating hospitals rather than actual changes. This reduces the precision of longitudinal analyses and should be addressed with a revised approach in possible future surveys. Third, certain concepts addressed in the IPCAF (e.g. multimodal strategies) are rather complex and might not always have been completely understood. This was addressed by several footnotes and explanations throughout the tool. Forth, some questions may have been perceived as potentially compromising and, despite the confidential nature of the survey, might have prompted wishful reporting. Fifth, after submission of responses to the NRC, participants could not retroactively correct errors or otherwise modify the entered data.

## Conclusion

Overall, IPC structures remain at a high level in German acute care hospitals. Despite some improvements, potentials for further improvement remain and illustrate the need for continued efforts in the field of IPC. To monitor developments and progress in the field of IPC, there could be merit in conducting similar assessments in Germany again in the future.

## Supplementary Information

Additional file 1

Additional file 2

## Data Availability

Not applicable.

## Abbreviations

CC: Core component
HAI: Healthcare-associated infection
IPC: Infection prevention and control
IPCAF: Infection Prevention and Control Assessment Framework
KISS: Krankenhaus-Infektions-Surveillance-System
NRC: National Reference Center for the Surveillance of Nosocomial Infections
WHO: World Health Organization
