# Supplementary material for: Assessing infection prevention and control structures in German hospitals after the COVID-19 pandemic using the WHO infection prevention and control assessment framework (IPCAF): results from 660 hospitals and comparison with a pre-pandemic survey
Source: Antimicrob Resist Infect Control. 2024 Sep 13;13:103. doi: 10.1186/s13756-024-01465-7 (PMC11396616; doi:10.1186/s13756-024-01465-7)
Supplement: Supplementary file 2 — Additional file 2 [file 13756_2024_1465_MOESM2_ESM.pdf]

Results of the Infection Prevention and Control Assessment Framework (IPCAF) in 660 German hospitals

Core component 1: Infection Prevention and Control (IPC) programme

Question/Answer	Number (%)
**1. Do you have an IPC programme?**	
No	38 (5.8)
Yes, without clearly defined objectives	237 (35.9)
Yes, with clearly defined objectives and annual activity plan	385 (58.3)
**2. Is the IPC programme supported by an IPC team comprising of IPC professionals?**	
No	34 (5.2)
Not a team, only an IPC focal person	32 (4.8)
Yes	594 (90.0)
**3. Does the IPC team have at least one full-time IPC professional or equivalent (nurse or doctor working 100% in IPC) available?**	
No IPC professional available	7 (1.1)
No, only a part-time IPC professional available	38 (5.8)
Yes, one per > 250 beds	144 (21.8)
Yes, one per ≤ 250 beds	471 (71.3)
**4. Does the IPC team or focal person have dedicated time for IPC activities?**	
No	80 (12.1)
Yes	580 (87.9)
**5. Does the IPC team include both doctors and nurses?**	
No	21 (3.2)
Yes	639 (96.8)
**6. Do you have an IPC committee actively supporting the IPC team?**	
No	12 (1.8)
Yes	648 (98.2)
**7. Are any of the following professional groups represented/included in the IPC committee?**	
**- Senior facility leadership (for example, administrative director, chief executive officer [CEO], medical director)**	
No	13 (2.0)
Yes	647 (98.0)
**- Senior clinical staff (for example, physician, nurse)**	
No	5 (0.8)
Yes	655 (99.2)
**- Facility management (for example, biosafety, waste, and those tasked with addressing water, sanitation, and hygiene [WASH])**	
No	108 (16.4)
Yes	552 (83.6)
**8. Do you have clearly defined IPC objectives (that is, in specific critical areas)?**	
No	30 (4.6)
Yes, IPC objectives only	125 (18.9)
Yes, IPC objectives and measurable outcome indicators (that is, adequate measures for improvement)	272 (41.2)
Yes, IPC objectives, measurable outcome indicators and set future targets	233 (35.3)

**9. Does the senior facility leadership show clear commitment and support for the IPC programme:**	
**- By an allocated budget specifically for the IPC programme (that is, covering IPC activities, including salaries)?**	
No	191 (28.9)
Yes	469 (71.1)
**- By demonstrable support for IPC objectives and indicators within the facility (for example, at executive level meetings, executive rounds, participation in morbidity and mortality meetings)?**	
No	200 (30.3)
Yes	460 (69.7)
**10. Does your facility have microbiological laboratory support (either present on or off site) for routine day-to-day use?**	
No	3 (0.5)
Yes, but not delivering results reliably (timely and of sufficient quality)	47 (7.1)
Yes, and delivering results reliably (timely and of sufficient quality)	610 (92.4)

Core component 2: Infection Prevention and Control (IPC) guidelines

Question	Number (%)
**1. Does your facility have the expertise (in IPC and/or infectious diseases) for developing or adapting guidelines?**	
No	43 (6.5)
Yes	617 (93.5)
**2. Does your facility have guidelines available for:**	
**- Standard precautions?**	
No	3 (0.5)
Yes	657 (99.5)
**- Hand hygiene?**	
No	2 (0.3)
Yes	658 (99.7)
**- Transmission-based precautions?**	
No	4 (0.6)
Yes	656 (99.4)
**- Outbreak management and preparedness?**	
No	6 (0.9)
Yes	654 (99.1)
**- Prevention of surgical site infection?**	
No	17 (2.6)
Yes	643 (97.4)
**- Prevention of vascular catheter-associated bloodstream infections?**	
No	14 (2.1)
Yes	646 (97.9)
**- Prevention of hospital-acquired pneumonia ([HAP]; all types of HAP, including (but not exclusively) ventilator-associated pneumonia)?**	
No	50 (7.6)
Yes	610 (92.4)
**- Prevention of catheter-associated urinary tract infections?**	
No	24 (3.6)
Yes	636 (96.4)
**- Prevention of transmission of multidrug-resistant (MDR) pathogens?**	
No	0 (0)
Yes	660 (100)
**- Disinfection and sterilization?**	
No	2 (0.3)

Yes	658 (99.7)
**- Health care worker protection and safety**	
No	12 (1.8)
Yes	648 (98.2)
**- Injection safety?**	
No	6 (0.9)
Yes	654 (99.1)
**- Waste management?**	
No	33 (5.0)
Yes	627 (95.0)
**- Antibiotic stewardship?**	
No	49 (7.4)
Yes	611 (92.6)
**3. Are the guidelines in your facility consistent with national/international guidelines (if they exist)?**	
No	18 (2.7)
Yes	642 (97.3)
**4. Is implementation of the guidelines adapted according to the local needs and resources while maintaining key IPC standards?**	
No	15 (2.3)
Yes	645 (97.7)
**5. Are frontline health care workers involved in both planning and executing the implementation of IPC guidelines in addition to IPC personnel?**	
No	52 (7.9)
Yes	608 (92.1)
**6. Are relevant stakeholders (for example, lead doctors and nurses, hospital managers, quality management) involved in the development and adaptation of the IPC guidelines in addition to IPC personnel?**	
No	58 (8.8)
Yes	602 (91.2)
**7. Do health care workers receive specific training related to new or updated IPC guidelines introduced in the facility?**	
No	45 (6.8)
Yes	615 (93.2)
**8. Do you regularly monitor the implementation of at least some of the IPC guidelines in your facility?**	
No	9 (1.4)
Yes	651 (98.6)

Core component 3: Infection Prevention and Control (IPC) education and training

Question	Number (%)
**1. Are there personnel with the IPC expertise (in IPC and/or infectious diseases) to lead IPC training?**	
No	10 (1.5)
Yes	650 (98.5)
**2. Are there additional non-IPC personnel with adequate skills to serve as trainers and mentors (for example, link nurses or doctors, champions)?**	
No	57 (8.6)
Yes	603 (91.4)

**3. How frequently do health care workers receive training regarding IPC in your facility?**	
Never or rarely	5 (0.8)
New employee orientation only for health care workers	1 (0.2)
New employee orientation and regular (at least annually) IPC training for health care workers offered but not mandatory	70 (10.5)
New employee orientation and regular (at least annually) mandatory IPC training for all health care workers	584 (88.5)
**4. How frequently do cleaners and other personnel directly involved in patient care receive training regarding IPC in your facility?**	
Never or rarely	10 (1.5)
New employee orientation only for other personnel	3 (0.5)
New employee orientation and regular (at least annually) training for other personnel offered but not mandatory	109 (16.5)
New employee orientation and regular (at least annually) mandatory IPC training for other personnel	538 (81.5)
**5. Does administrative and managerial staff receive general training regarding IPC in your facility?**	
No	191 (28.9)
Yes	469 (71.1)
**6. How are health care workers and other personnel trained?**	
No training available	3 (0.5)
Using written information and/or oral instruction and/or e-learning only	373 (56.5)
Includes additional interactive training sessions (for example, simulation and/or bedside training)	284 (43.0)
**7. Are there periodic evaluations of the effectiveness of training programmes (for example, hand hygiene audits, other checks on knowledge)?**	
No	73 (11.1)
Yes, but not regularly	225 (34.1)
Yes, regularly (at least annually)	362 (54.8)
**8. Is IPC training integrated in the clinical practice and training of other specialties (for example, training of surgeons involves aspects of IPC)?**	
No	125 (18.9)
Yes, in some disciplines	266 (40.3)
Yes, in all disciplines	269 (40.8)
**9. Is there specific IPC training for patients or family members to minimize the potential for health care-associated infections (for example, immunosuppressed patients, patients with invasive devices, patients with multidrug-resistant infections)?**	
No	406 (61.5)
Yes	254 (38.5)
**10. Is ongoing development/education offered for IPC staff (for example, by regularly attending conferences, courses)?**	
No	14 (2.1)
Yes	646 (97.9)

Core component 4: Health care-associated infection (HAI) surveillance

Question	Number (%)
**1. Is surveillance a defined component of your IPC programme?**	
No	9 (1.4)

Yes	651 (98.6)
**2. Do you have personnel responsible for surveillance activities?**	
No	10 (1.5)
Yes	650 (98.5)
**3. Have the professionals responsible for surveillance activities been trained in basic epidemiology, surveillance and IPC (that is, capacity to oversee surveillance methods, data management and interpretation)?**	
No	10 (1.5)
Yes	650 (98.5)
**4. Do you have informatics/IT support to conduct your surveillance (for example, equipment, mobile technologies, electronic health records)?**	
No	70 (10.6)
Yes	590 (89.4)
**5. Do you go through a prioritization exercise to determine the HAIs to be targeted for surveillance according to the local context (that is, identifying infections that are major causes of morbidity and mortality in the facility)?**	
No	79 (12.0)
Yes	581 (88.0)
**6. In your facility is surveillance conducted for:**	
**- Surgical site infections?**	
No	38 (5.8)
Yes	622 (94.2)
**- Device-associated infections (for example, catheter-associated urinary tract infections, central line-associated bloodstream infections, peripheral-line associated bloodstream infections, ventilator-associated pneumonia)?**	
No	75 (11.4)
Yes	585 (88.6)
**- Clinically-defined infections (for example, definitions based only on clinical signs or symptoms in the absence of microbiological testing)?**	
No	278 (42.1)
Yes	382 (57.9)
**- Colonization or infections caused by multidrug-resistant pathogens according to your local epidemiological situation?**	
No	20 (3.0)
Yes	640 (97.0)
**- Local priority epidemic-prone infections (for example, norovirus, influenza, tuberculosis [TB], severe acute respiratory syndrome [SARS], Ebola, Lassa fever)?**	
No	92 (13.9)
Yes	568 (86.1)
**- Infections in vulnerable populations (for example, neonates, intensive care unit, immunocompromised, burn patients)?**	
No	91 (13.8)
Yes	569 (86.2)
**- Infections that may affect health care workers in clinical, laboratory, or other settings (for example, hepatitis B or C, human immunodeficiency virus [HIV], influenza)?**	
No	370 (56.1)
Yes	290 (43.9)
**7. Do you regularly evaluate if your surveillance is in line with the current needs and priorities of your facility?**	
No	85 (12.9)
Yes	575 (87.1)

**8. Do you use reliable surveillance case definitions (defined numerator and denominator according to international definitions [e.g. CDC NHSN/ECDC] or if adapted, through an evidence-based adaptation process and expert consultation?**	
No	69 (10.5)
Yes	591 (89.5)
**9. Do you use standardized data collection methods (for example, active prospective surveillance) according to international surveillance protocols (for example, CDC NHSN/ECDC) or if adapted, through an evidence-based adaptation process and expert consultation?**	
No	97 (14.7)
Yes	563 (85.3)
**10. Do you have processes in place to regularly review data quality (for example, assessment of case report forms, review of microbiology results, denominator determination, etc.)?**	
No	113 (17.1)
Yes	547 (82.9)
**11. Do you have adequate microbiology and laboratory capacity to support surveillance?**	
No	7 (1.1)
Yes, can differentiate gram-positive/negative strains but cannot identify pathogens	3 (0.5)
Yes, can reliably identify pathogens (for example, isolate identification) in a timely manner	15 (2.3)
Yes, can reliably identify pathogens and antimicrobial drug resistance patterns (that is, susceptibilities) in a timely manner	635 (96.1)
**12. Are surveillance data used to make tailored unit/facility-based plans for the improvement of IPC practices?**	
No	42 (6.4)
Yes	618 (93.6)
**13. Do you analyze antimicrobial drug resistance on a regular basis (for example, quarterly/half-yearly/annually)?**	
No	40 (6.1)
Yes	620 (93.9)
**14. Do you regularly (for example, quarterly/half-yearly/annually) feedback up-to-date surveillance information to:**	
**- Frontline health care workers (doctors/nurses)?**	
No	53 (8.0)
Yes	607 (92.0)
**- Clinical leaders/heads of department**	
No	36 (5.5)
Yes	624 (94.5)
**- IPC committee**	
No	8 (1.2)
Yes	652 (98.8)
**- Non-clinical management/administration (chief executive officer/chief financial officer)?**	
No	82 (12.4)
Yes	578 (87.6)
**15. How do you feedback up-to-date surveillance information? (at least annually)**	
No feedback	4 (0.6)
By written/oral information only	185 (28.0)
By presentation and interactive problem-orientated solution finding	471 (71.4)

Core component 5: Multimodal strategies for implementation of infection prevention and control (IPC) interventions

Question	Number (%)
**1. Do you use multimodal strategies to implement IPC interventions?**	
No	98 (14.8)
Yes	562 (85.2)
**2. Do your multimodal strategies include any or all of the following elements:**	
**- System change**	
Element not included in multimodal strategies	143 (21.7)
Interventions to ensure the necessary infrastructure and continuous availability of supplies are in place	216 (32.7)
Interventions to ensure the necessary infrastructure and continuous availability of supplies are in place and addressing ergonomics and accessibility, such as the best placement of central venous catheter set and tray	301 (45.6)
**- Education and training**	
Element not included in multimodal strategies	44 (6.7)
Written information and/or oral instruction and/or e-learning only	355 (53.8)
Additional interactive training sessions (includes simulation and/or bedside training)	261 (39.5)
**- Monitoring and feedback**	
Element not included in multimodal strategies	63 (9.5)
Monitoring compliance with process or outcome indicators (for example, audits of hand hygiene or catheter practices)	186 (28.2)
Monitoring compliance and providing timely feedback of monitoring results to health care workers and key players	411 (62.3)
**- Communications and reminders**	
Element not included in multimodal strategies	125 (18.9)
Reminders, posters, or other advocacy/awareness-raising tools to promote the intervention	352 (53.4)
Additional methods/initiatives to improve team communication across units and disciplines (for example, by establishing regular case conferences and feedback rounds)	183 (27.7)
**- Safety climate and culture change**	
Element not included in multimodal strategies	271 (41.1)
Managers/leaders show visible support and act as champions and role models, promoting an adaptive approach and strengthening a culture that supports IPC, patient safety and quality	271 (41.1)
Additionally, teams and individuals are empowered so that they perceive ownership of the intervention (for example, by participatory feedback rounds)	118 (17.8)
**3. Is a multidisciplinary team used to implement IPC multimodal strategies?**	
No	199 (30.2)
Yes	461 (69.8)
**4. Do you regularly link to colleagues from quality improvement and patient safety to develop and promote IPC multimodal strategies?**	
No	193 (29.2)
Yes	467 (70.8)
**5. Do these strategies include bundles or checklists?**	
No	106 (16.1)
Yes	554 (83.9)

Core component 6: Monitoring/audit of IPC practices and feedback

Question	Number (%)
**1. Do you have trained personnel responsible for monitoring/audit of IPC practices and feedback?**	
No	20 (3.0)
Yes	640 (97.0)
**2. Do you have a well-defined monitoring plan with clear goals, targets and activities (including tools to collect data in a systematic way)?**	
No	143 (21.7)
Yes	517 (78.3)
**3. Which processes and indicators do you monitor in your facility?** (Tick all that apply)	
None	2 (0.3)
Hand hygiene compliance (using the WHO hand hygiene observation tool or equivalent)	605 (91.7)
Intravascular catheter insertion and/or care	493 (74.7)
Wound dressing change	500 (75.8)
Transmission-based precautions and isolation to prevent the spread of multidrug resistant organisms (MDRO)	634 (96.1)
Cleaning of the ward environment	605 (91.7)
Disinfection and sterilization of medical equipment/instruments	594 (90.0)
Consumption/usage of alcohol-based handrub or soap	651 (98.6)
Consumption/usage of antimicrobial agents	634 (96.1)
Waste management	426 (64.5)
**4. How frequently is the WHO Hand Hygiene Self-Assessment Framework Survey undertaken?**	
Never	411 (62.3)
Periodically, but no regular schedule	122 (18.5)
At least annually	127 (19.2)
**5. Do you feedback auditing reports (for example, feedback on hand hygiene compliance data or other processes) on the state of the IPC activities/performance?** (Tick all that apply)	
No reporting	6 (0.9)
Yes, within the IPC team	648 (98.2)
Yes, to department leaders and managers in the areas being audited	646 (97.9)
Yes, to frontline health care workers	624 (94.5)
Yes, to the IPC committee or quality of care committees or equivalent	592 (89.7)
Yes, to hospital management and senior administration	580 (87.9)
**6. Is the reporting of monitoring data undertaken regularly (at least annually)?**	
No	25 (3.8)
Yes	635 (96.2)
**7. Are monitoring and feedback of IPC processes and indicators performed in a "blame-free" institutional culture aimed at improvement and behavioural change?**	
No	26 (3.9)
Yes	634 (96.1)
**8. Do you assess safety cultural factors in your facility (for example, by using other surveys such as HSOPSC, SAQ, PSCHO, HSC)**	
No	499 (75.6)
Yes	161 (24.4)

Core component 7: Workload, staffing and bed occupancy

Question	Number (%)
**1. Are appropriate staffing levels assessed in your facility according to patient workload using national standards or a standard staffing needs assessment tool such as the WHO Workload indicators of staffing need method?**	
No	107 (16.2)
Yes	553 (83.8)
**2. Is an agreed (that is, WHO or national) ratio of health care workers to patients maintained across your facility?**	
No	48 (7.3)
Yes, for staff in less than 50% of units	43 (6.5)
Yes, for staff in more than 50% of units	215 (32.6)
Yes, for all health care workers in the facility	354 (53.6)
**3. Is a system in place in your facility to act on the results of the staffing needs assessments when staffing levels are deemed to be too low?**	
No	107 (16.2)
Yes	553 (83.8)
**4. Is the design of wards in your facility in accordance with international standards regarding bed capacity?**	
No	42 (6.4)
Yes, but only in certain departments	215 (32.6)
Yes, for all departments (including emergency department and pediatrics)	403 (61.0)
**5. Is bed occupancy in your facility kept to one patient per bed?**	
No	10 (1.5)
Yes, but only in certain departments	44 (6.7)
Yes, for all units (including emergency departments and pediatrics)	606 (91.8)
**6. Are patients in your facility placed in beds standing in the corridor outside of the room (including beds in the emergency department)?**	
Yes, more frequently than twice a week	70 (10.6)
Yes, less frequently than twice a week	104 (15.8)
No	486 (73.6)
**7. Is adequate spacing of > 1 meter between patient beds ensured in your facility?**	
No	16 (2.4)
Yes, but only in certain departments	134 (20.3)
Yes, for all departments (including emergency department and pediatrics)	510 (77.3)
**8. Is a system in place in your facility to assess and respond when adequate bed capacity is exceeded?**	
No	18 (2.7)
Yes, this is the responsibility of the head of department	237 (35.9)
Yes, this is the responsibility of the hospital administration/management	405 (61.4)

Core component 8: Built environment, materials and equipment for IPC at the facility level

Question	Number (%)
**1. Are water services available at all times and of sufficient quantity for all uses (for example, hand washing, drinking, personal hygiene, medical activities, sterilization, decontamination, cleaning and laundry)?**	
No, available on average < 5 days per week	0 (0)
Yes, available on average ≥ 5 days per week or every day but not of sufficient quantity	3 (0.5)

Yes, every day and of sufficient quantity	657 (99.5)
**2. Is a reliable safe drinking water station present and accessible for staff, patients and families at all times and in all locations/wards?**	
No, not available	3 (0.5)
Sometimes, or only in some places or not available for all users	10 (1.5)
Yes, accessible at all times and for all wards/groups	647 (98.0)
**3. Are functioning hand hygiene stations (that is, alcohol-based handrub solution or soap and water and clean single-use towels) available at all points of care?**	
No, not present	5 (0.8)
Yes, stations present, but supplies are not reliably available	5 (0.8)
Yes, with reliably available supplies	650 (98.4)
**4. In your facility, are ≥ 4 toilets or improved latrines available for outpatient settings or ≥ 1 per 20 users for inpatient settings?**	
Less than required number of toilets or latrines available and functioning	12 (1.8)
Sufficient number present but not all functioning	1 (0.2)
Sufficient number present and functioning	647 (98.0)
**5. In your health care facility, is sufficient energy/power supply available at day and night for all uses (for example, pumping and boiling water, sterilization and decontamination, incineration or alternative treatment technologies, electronic medical devices, general lighting of areas where health care procedures are performed to ensure safe provision of health care and lighting of toilet facilities and showers)?**	
No	0 (0)
Yes, sometimes or only in some of the mentioned areas	0 (0)
Yes, always and in all mentioned areas	660 (100)
**6. Is functioning environmental ventilation (natural or mechanical) available in patient care areas?**	
No	5 (0.8)
Yes	655 (99.2)
**7. For floors and horizontal work surfaces, is there an accessible record of cleaning, signed by the cleaners each day?**	
No record of floors and surfaces being cleaned	192 (29.1)
Record exists, but is not completed and signed daily or is outdated	113 (17.1)
Yes, record completed and signed daily	355 (53.8)
**8. Are appropriate and well-maintained materials for cleaning (for example, detergent, mops, buckets, etc.) available?**	
No materials available	0 (0)
Yes, available but not well maintained	9 (1.4)
Yes, available and well-maintained	651 (98.6)
**9. Do you have single patient rooms or rooms for cohorting patients with similar pathogens if the number of isolation rooms is insufficient (for example, TB, measles, cholera, Ebola, SARS)?**	
No	7 (1.1)
No single rooms but rather rooms suitable for patient cohorting available	47 (7.1)
Yes, single rooms are available	606 (91.8)
**10. Is PPE available at all times and in sufficient quantity for all uses for all health care workers?**	
No	0 (0)
Yes, but not continuously available in sufficient quantities	1 (0.2)
Yes, continuously available in sufficient quantities	659 (99.8)

**11. Do you have functional waste collection containers for non-infectious (general) waste, infectious waste and, sharps waste in close proximity to all waste generation points?**	
No bins or separate sharps disposal	0 (0)
Separate bins present but lids missing or more than 3/4 full; only two bins (instead of three); or bins at some but not all waste generation points	22 (3.3)
Yes	638 (96.7)
**12. Is a functional burial pit/fenced waste dump or municipal pick-up available for disposal of non-infectious (non-hazardous/general waste)?**	
No pit or other disposal method used	7 (1.1)
Pit in facility but insufficient dimensions; pits/dumps overfilled or not fenced/locked; or irregular municipal waste pick up	8 (1.2)
Yes	645 (97.7)
**13. Is an incinerator or alternative treatment technology for the treatment of infectious and sharp waste (for example, an autoclave) present (either present on or off site and operated by a licensed waste management service), functional and of a sufficient capacity?**	
No, none present	38 (5.8)
Present, but not functional	0 (0)
Yes	622 (94.2)
**14. Is a wastewater treatment system (for example, septic tank followed by drainage pit) present (either on or off site) and functioning reliably?**	
No, not present	34 (5.2)
Yes, but not functioning reliably	0 (0)
Yes and functioning reliably	626 (94.8)
**15. Does your health care facility provide a dedicated decontamination area and/or sterile supply department (either present on or off site and operated by a licensed decontamination management service) for the decontamination and sterilization of medical devices and other items/equipment?**	
No, not present	25 (3.8)
Yes, but not functioning reliably	7 (1.1)
Yes and functioning reliably	628 (95.1)
**16. Do you reliably have sterile and disinfected equipment ready for use?**	
No, available on average < five days per week	0 (0)
Yes, available on average ≥ five days per week or every day, but not of sufficient quantity	5 (0.8)
Yes, available every day and of sufficient quantity	655 (99.2)
**17. Are disposable items available when necessary? (for example, injection safety devices, examination gloves)**	
No, not available	0 (0)
Yes, but only sometimes available	2 (0.3)
Yes, continuously available	658 (99.7)
